# Impaired Photorespiratory Metabolism Underlies the Decline in CO_2_ Assimilation Induced by Alternative Oxidase Inhibition in *Rumex* K-1 Leaves

**DOI:** 10.3390/plants15172737

**Published:** 2026-09-07

**Authors:** Xin Zhong, Shuhao Li, Litao Zhang

**Affiliations:** 1 Institute of Marine Science and Technology, Shandong University, Qingdao 266237, China; zhongxin0401@sdu.edu.cn; 2 Southern Zhejiang Key Laboratory of Crop Breeding, Wenzhou Academy of Agricultural Sciences, Wenzhou 325006, China; 3 Laboratory of Experimental Marine Biology, Institute of Oceanology, Chinese Academy of Sciences, Qingdao 266000, China

**Keywords:** alternative oxidase (AOX), photorespiration, glycine–serine metabolism, mitochondrial redox homeostasis, photosynthetic carbon assimilation

## Abstract

Photosynthetic carbon assimilation under photorespiratory conditions requires tight coordination between chloroplast electron transport and mitochondrial redox metabolism, yet the contribution of mitochondrial alternative oxidase (AOX) remains unresolved. Using 1 mM salicylhydroxamic acid (SHAM) to inhibit the AOX pathway in *Rumex* K-1 leaves, we investigated how mitochondrial alternative respiration contributes to carbon assimilation. AOX inhibition imposed a non-stomatal limitation on CO_2_ assimilation and reduced photosystem II (PSII) electron transport. However, low O_2_ or elevated CO_2_ alleviated the decline in CO_2_ assimilation while PSII photochemistry remained depressed, and AOX inhibition reduced apparent *V*_cmax_ without significantly affecting *J*_max_, indicating that the primary constraint lay downstream of PSII in photorespiratory carbon metabolism. Electron flux through PSII and the electron fluxes supporting the photosynthetic carbon reduction and photorespiratory carbon oxidation cycles all decreased under SHAM treatment, indicating reduced PSII electron transport and electron use associated with carbon assimilation and photorespiration. AOX inhibition caused glycine accumulation and increased the Gly/Ser ratio under illumination but not in darkness, indicating restricted mitochondrial glycine-to-serine conversion during photorespiration. Together, these responses suggest that AOX-dependent ubiquinol oxidation helps sustain mitochondrial NADH reoxidation and NAD^+^ regeneration, thereby supporting glycine-to-serine conversion and photorespiratory carbon recycling.

## 1. Introduction

Photosynthetic carbon assimilation supplies the organic carbon required for plant growth and represents the principal entry point of carbon into terrestrial ecosystems [1,2]. Efficient photosynthesis requires light harvesting and electron transport to be balanced with the capacity of carbon assimilation and other electron-consuming processes to utilize the ATP and NADPH generated by the light reactions [3,4]. This balance can be disrupted by excess light, abrupt increases in irradiance, CO_2_ limitation, or other environmental constraints, causing over-reduction of photosynthetic electron carriers and stromal electron acceptors [5,6]. The resulting imbalance between excitation pressure and electron-utilization capacity enhances reactive oxygen species (ROS) formation and photooxidative stress; in photosystem II (PSII), photoinhibition ensues when photodamage outpaces repair [7,8,9]. Plants therefore deploy an integrated network of photoprotective and repair processes—including non-photochemical quenching, alternative electron sinks, antioxidant systems, and the PSII repair cycle—to dissipate excess excitation energy, provide outlets for surplus electrons, detoxify ROS, and restore damaged PSII complexes [4,5,10].

Although many canonical photoprotective mechanisms operate within chloroplasts, the maintenance of photosynthetic performance in intact leaves depends on metabolic coordination across the whole cell [11,12,13]. Chloroplasts, mitochondria, peroxisomes, and the cytosol communicate metabolically through redox-active metabolite shuttles, including the malate–oxaloacetate shuttle [13]. Mitochondrial electron transport can thereby indirectly influence chloroplast redox poise by oxidizing reducing equivalents generated through respiratory and photorespiratory metabolism and by sustaining metabolite exchange between organelles. In pea mesophyll protoplasts, pharmacological inhibition of the mitochondrial alternative oxidase pathway decreased the light activation of three Calvin–Benson cycle enzymes and chloroplastic NADP-dependent malate dehydrogenase (NADP-MDH), suggesting that mitochondrial metabolism can feed back on chloroplast redox regulation and carbon assimilation [14]. In C_3_ leaves, photorespiration is a major component of this interorganellar coupling: ribulose-1,5-bisphosphate carboxylase/oxygenase (Rubisco) oxygenation generates 2-phosphoglycolate, which is recycled to 3-phosphoglycerate through reactions distributed across chloroplasts, peroxisomes, and mitochondria. In the *Nicotiana sylvestris* CMSII mutant, which lacks functional mitochondrial complex I, steady-state photosynthesis was reduced at atmospheric CO_2_, whereas high CO_2_ or low O_2_ alleviated the inhibition, showing that complex I is required for optimal photosynthetic performance under photorespiratory conditions [11]. Compartment-resolved biosensor measurements in *Arabidopsis thaliana* further showed that inhibiting glycine (Gly) decarboxylation abolished the light-induced increases in stromal NADPH and the stromal NADH/NAD^+^ ratio, suggesting that mitochondrial photorespiratory metabolism contributes to intercompartmental redox balancing through metabolite shuttles [13].

The ubiquinone/ubiquinol pool forms a major branch point in the plant mitochondrial electron transport chain, receiving electrons from multiple dehydrogenases and partitioning them between the cytochrome and alternative oxidase (AOX) pathways [15,16]. Whereas electron transfer through the cytochrome pathway (complex III–cytochrome c–complex IV) contributes to proton-motive force generation and ATP synthesis, AOX directly couples ubiquinol oxidation to the reduction of O_2_ to water without proton translocation at this branch [17,18]. Although this lowers the energy conserved during respiration, AOX provides an alternative route for ubiquinol oxidation when electron input exceeds the oxidative capacity of the cytochrome pathway or the mitochondrial ubiquinone pool becomes highly reduced. AOX is therefore not simply a non-energy-conserving bypass, but a regulated respiratory branch that buffers the redox state of the ubiquinone pool and supports cellular redox balance [16,19,20,21].

Genetic and physiological evidence links AOX activity to the maintenance of photosynthetic performance. In *Arabidopsis* mutants defective in cyclic electron flow around photosystem I (PSI), chloroplast over-reduction was accompanied by increased activities of reductant-export enzymes, greater AOX protein abundance, and enhanced cyanide-resistant respiration; high light similarly induced AOX accumulation in the wild type, consistent with a role for AOX in facilitating the oxidation of reducing equivalents exported from chloroplast metabolism [22]. *Arabidopsis aox1a* mutants also exhibited lower CO_2_ assimilation under elevated CO_2_, together with reduced PSII quantum yield, maximum electron transport rate, estimated rate of ribulose-1,5-bisphosphate (RuBP) regeneration, and malate–oxaloacetate (Mal/OAA) shuttle activity [23]. Studies using partial inhibition of complex III further showed that *aox1a* plants failed to sustain chloroplast electron transport and non-photochemical quenching, exhibited disturbed NAD(P)H and ascorbate redox balance, and, under high light, developed greater chloroplast over-reduction and lower PSII electron transport than the wild type [24,25]. Together, these findings support a role for AOX in coordinating mitochondrial reductant oxidation with chloroplast redox balance, photosynthetic electron transport, and carbon assimilation.

The relationship between AOX and photosynthetic metabolism is particularly relevant in C_3_ plants because photorespiration imposes a substantial demand on mitochondrial capacity for reductant oxidation and redox balancing. During the mitochondrial phase of photorespiration, glycine is converted to serine (Ser) through the sequential activities of the glycine decarboxylase complex (GDC) and serine hydroxymethyltransferase (SHMT), with GDC generating NADH and thereby linking photorespiratory metabolism to mitochondrial redox status. Recent studies indicate that the contribution of AOX to photosynthetic performance appears to depend strongly on photorespiratory activity. Inhibition of AOX alters mitochondrial redox balance and glycine-derived reductant metabolism under photorespiratory conditions, whereas the effects of AOX inhibition become less pronounced when photorespiration is suppressed [26]. Moreover, integrated analyses of metabolic fluxes and metabolite pools have shown that glycine availability can constrain photosynthetic acclimation during transitions in photorespiratory activity, highlighting the role of Gly/Ser metabolism in regulating dynamic carbon assimilation [27].

The hybrid sorrel *Rumex* K-1 (*R. patientia* × *R. tianschanicus*) has been used as a non-model C_3_ system to investigate metabolic coordination between chloroplasts and mitochondria in illuminated leaves. Previous studies showed that AOX inhibition compromises photoprotection in *Rumex* K-1 leaves under both low and high irradiance; under high irradiance, it further enhances PSI acceptor-side over-reduction, restricts linear electron flow and the induction of non-photochemical quenching, and increases H_2_O_2_ accumulation [12,28]. However, although these studies established an important role for AOX in photoprotection, the physiological basis of the decline in CO_2_ assimilation following AOX inhibition remains unclear in this species.

To address this question, we inhibited AOX with salicylhydroxamic acid (SHAM) and used a combination of physiological approaches to examine its effects on CO_2_ assimilation, photosynthetic electron allocation, and photorespiratory Gly/Ser metabolism in *Rumex* K-1 leaves. We specifically tested whether the decline in CO_2_ assimilation was associated primarily with downstream carbon and photorespiratory metabolism rather than originating from stomatal limitation or an initial defect in PSII photochemistry. This study extends current understanding of AOX-dependent photoprotection by clarifying the physiological links among mitochondrial alternative respiration, photosynthetic electron utilization, photorespiratory Gly/Ser metabolism, and carbon assimilation.

## 2. Results

**Figure 1:**
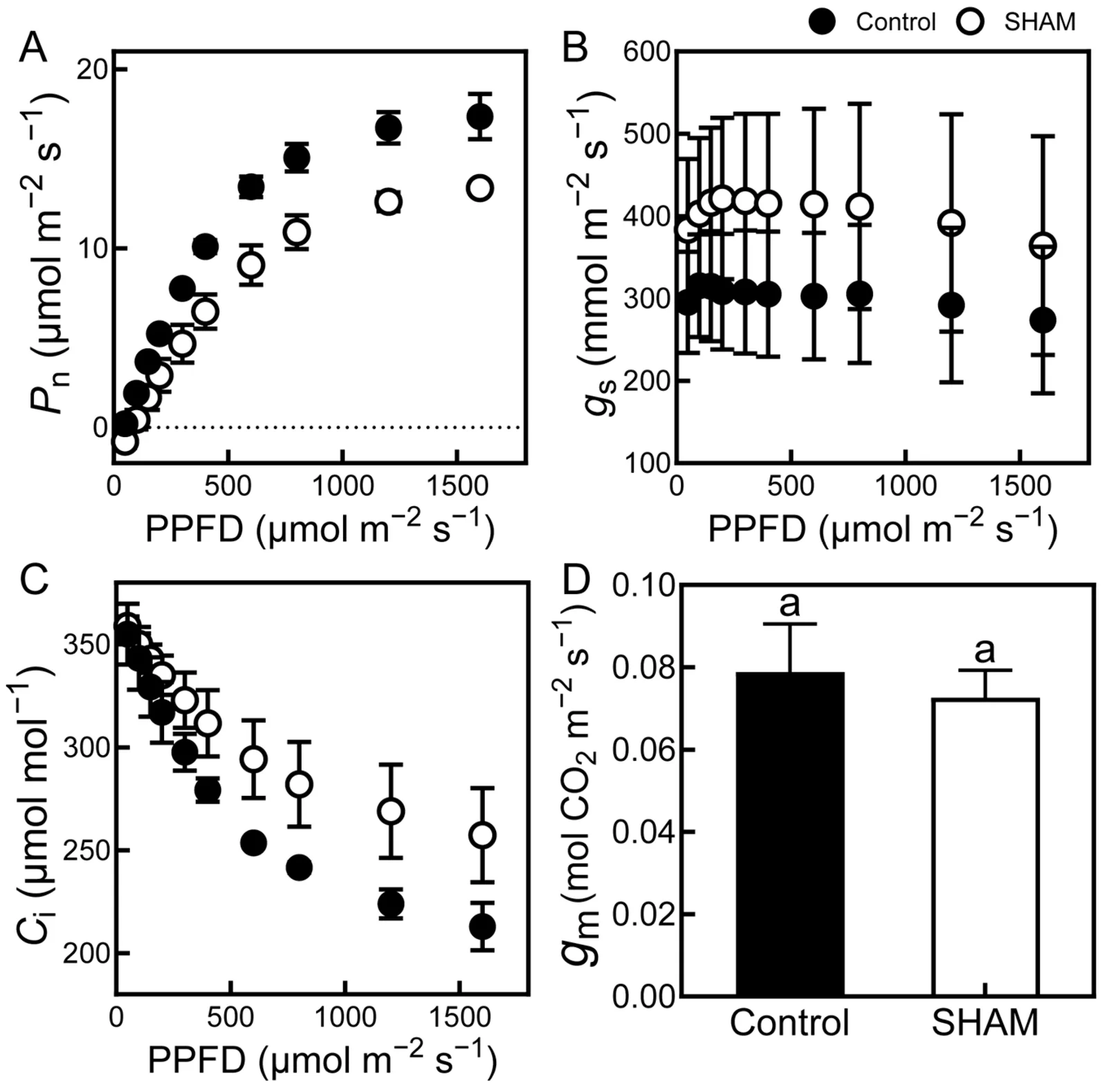
Light-response characteristics of photosynthesis following inhibition of the alternative oxidase (AOX) pathway in *Rumex* K-1 leaves. (**A**–**C**) Responses of net photosynthetic rate [*P*_n_; (**A**)], stomatal conductance [*g*_s_; (**B**)], and intercellular CO_2_ concentration [*C*_i_; (**C**)] to increasing PPFD in control and SHAM-treated leaves. (**D**) Mesophyll conductance (*g*_m_) in control and SHAM-treated leaves. Filled and open circles in panels (**A**–**C**) indicate control and SHAM-treated leaves, respectively; bars in panel (**D**) represent the corresponding treatment means. Data are presented as means ± standard errors (SE) (*n* = 5). The identical lowercase letters above the bars in panel (**D**) indicate no significant difference between control and SHAM-treated leaves (*p* = 0.656).

Inhibition of the AOX pathway altered the photosynthetic responses of *Rumex* K-1 leaves to increasing irradiance (Figure 1). Across the photosynthetic photon flux density (PPFD) gradient, net CO_2_ assimilation rate (*P*_n_) increased in both control and SHAM-treated leaves, but remained consistently lower in SHAM-treated leaves, with the difference becoming more pronounced at moderate to high PPFD (Figure 1A). In contrast, stomatal conductance (*g*_s_) varied relatively little across the light gradient and showed no clear difference between treatments (Figure 1B). Although intercellular CO_2_ concentration (*C*_i_) decreased with increasing PPFD in both treatments, SHAM-treated leaves maintained higher *C*_i_ than control leaves under moderate- and high-light conditions (Figure 1C). Mesophyll conductance (*g*_m_) did not differ significantly between control and SHAM-treated leaves (Figure 1D; *p* = 0.656). The reduction in *P*_n_, together with the absence of a corresponding decrease in *g*_s_, the higher *C*_i_, and the unchanged *g*_m_, indicates that AOX inhibition predominantly limited photosynthesis through non-stomatal processes.

**Figure 2:**
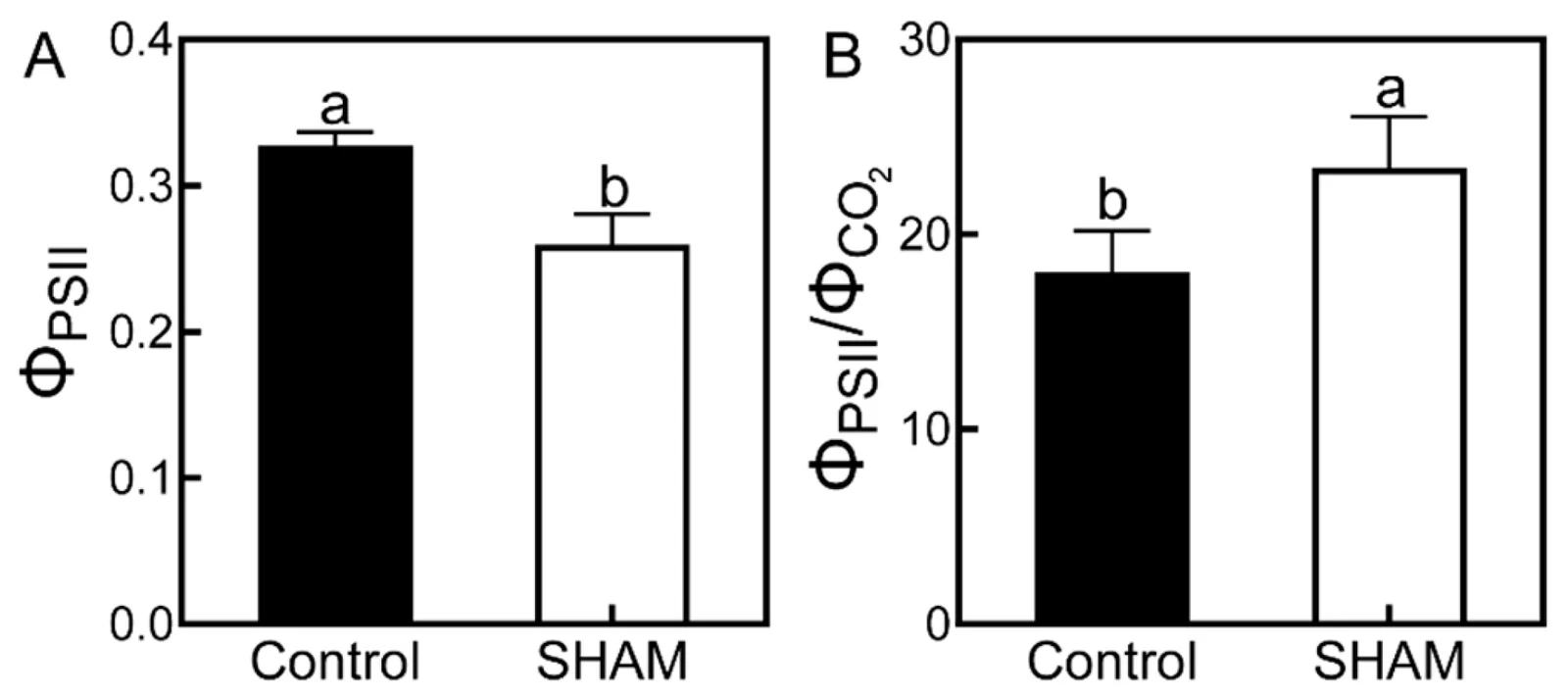
Effects of AOX pathway inhibition on the effective quantum yield of photosystem II and its ratio to the quantum yield of CO_2_ assimilation in *Rumex* K-1 leaves. (**A**) The effective quantum yield of photosystem II (Φ_PSII_) in control and SHAM-treated leaves. (**B**) The ratio of Φ_PSII_ to the quantum yield of CO_2_ assimilation (Φ_CO2_) in control and SHAM-treated leaves. Data are presented as means ± SE (*n* = 5). Different lowercase letters indicate significant differences between treatments at *p* < 0.05.

**Figure 3:**
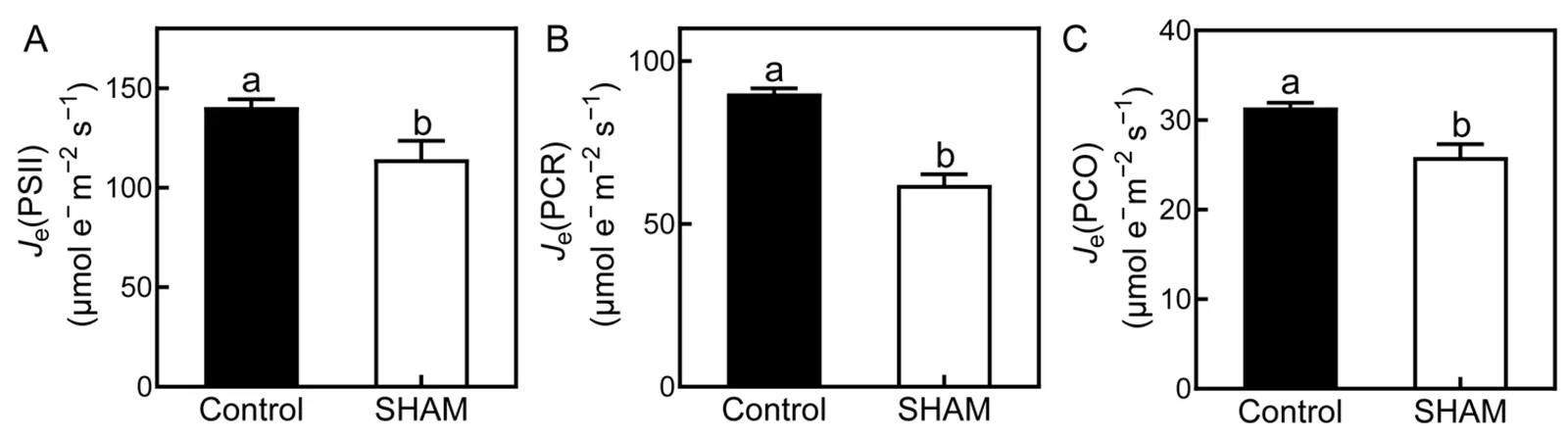
Effects of AOX pathway inhibition on photosynthetic electron fluxes in *Rumex* K-1 leaves. Total electron flux through photosystem II [*J*_e_(PSII); (**A**)], electron flux associated with the photosynthetic carbon reduction cycle [*J*_e_(PCR); (**B**)], and electron flux associated with the photorespiratory carbon oxidation cycle [*J*_e_(PCO); (**C**)] were estimated in control and SHAM-treated leaves. Black and white bars represent the control and SHAM treatments, respectively. Data are presented as means ± SE (*n* = 5). Different lowercase letters indicate significant differences between treatments at *p* < 0.05.

SHAM-treated leaves exhibited a significantly lower effective quantum yield of photosystem II (Φ_PSII_) but a higher ratio of Φ_PSII_ to the apparent quantum yield of net CO_2_ assimilation (Φ_CO2_) than control leaves (Figure 2A,B; *p* < 0.05). Analysis of photosynthetic electron fluxes further showed that the estimated total PSII electron flux [*J*_e_(PSII)] and the estimated electron fluxes associated with the photosynthetic carbon reduction cycle [*J*_e_(PCR)] and the photorespiratory carbon oxidation cycle [*J*_e_(PCO)] were significantly lower under SHAM treatment (Figure 3A–C; *p* < 0.05). These results indicate that SHAM treatment reduced PSII operating efficiency and the estimated electron fluxes associated with both photosynthetic carbon reduction and photorespiratory carbon oxidation.

**Figure 4:**
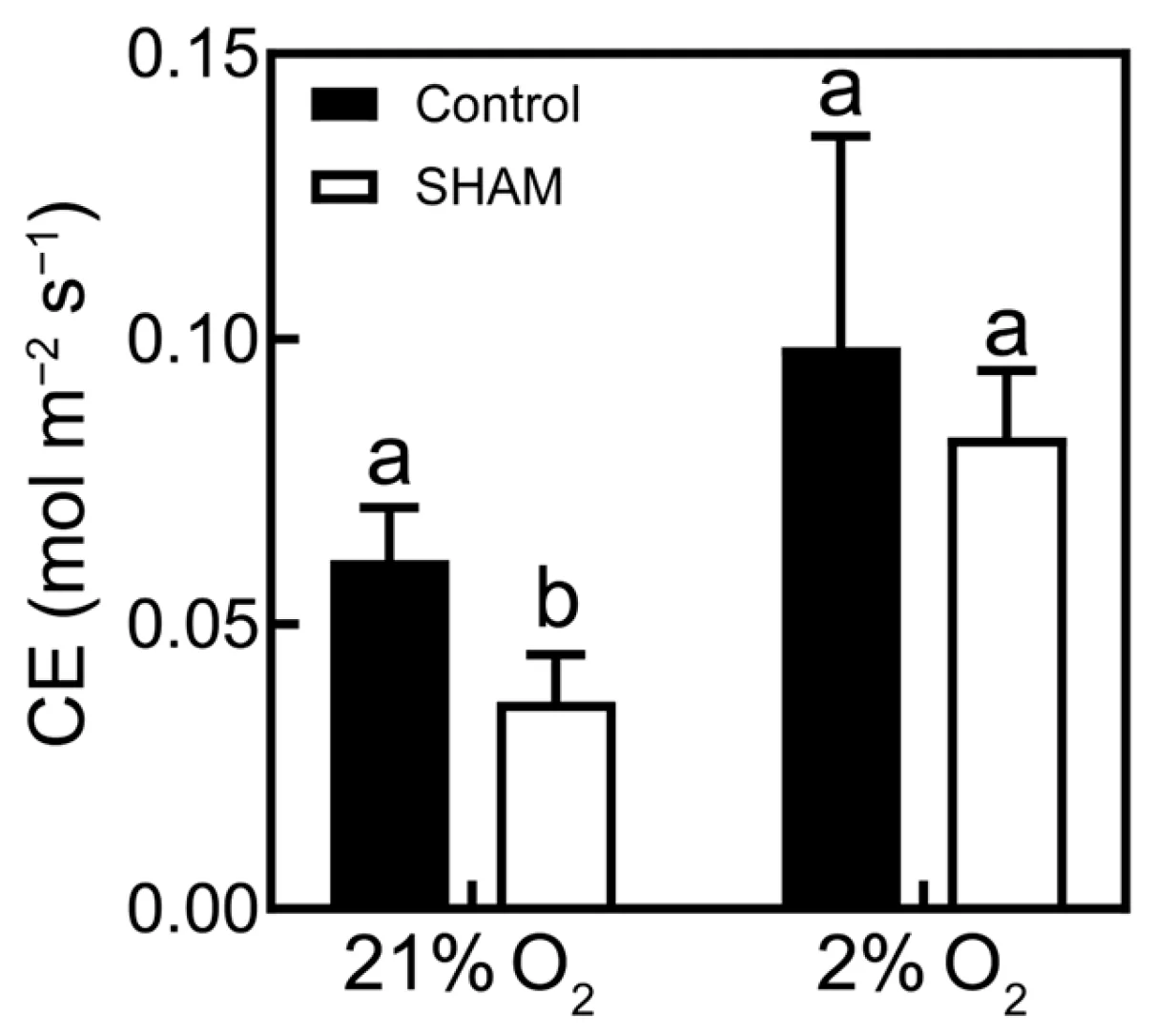
Effects of AOX pathway inhibition on carboxylation efficiency in *Rumex* K-1 leaves under ambient and low-O_2_ conditions. CE was determined in control and SHAM-treated leaves under 21% and 2% O_2_. Black and white bars represent the control and SHAM treatments, respectively. Data are presented as means ± SE (*n* = 5). Different lowercase letters indicate significant differences between treatments within each O_2_ condition at *p* < 0.05.

Under 21% O_2_, apparent carboxylation efficiency (CE) was significantly lower in SHAM-treated leaves than in control leaves (Figure 4; *p* < 0.05). By contrast, no significant difference in CE was detected between treatments under 2% O_2_ (Figure 4).

**Figure 5:**
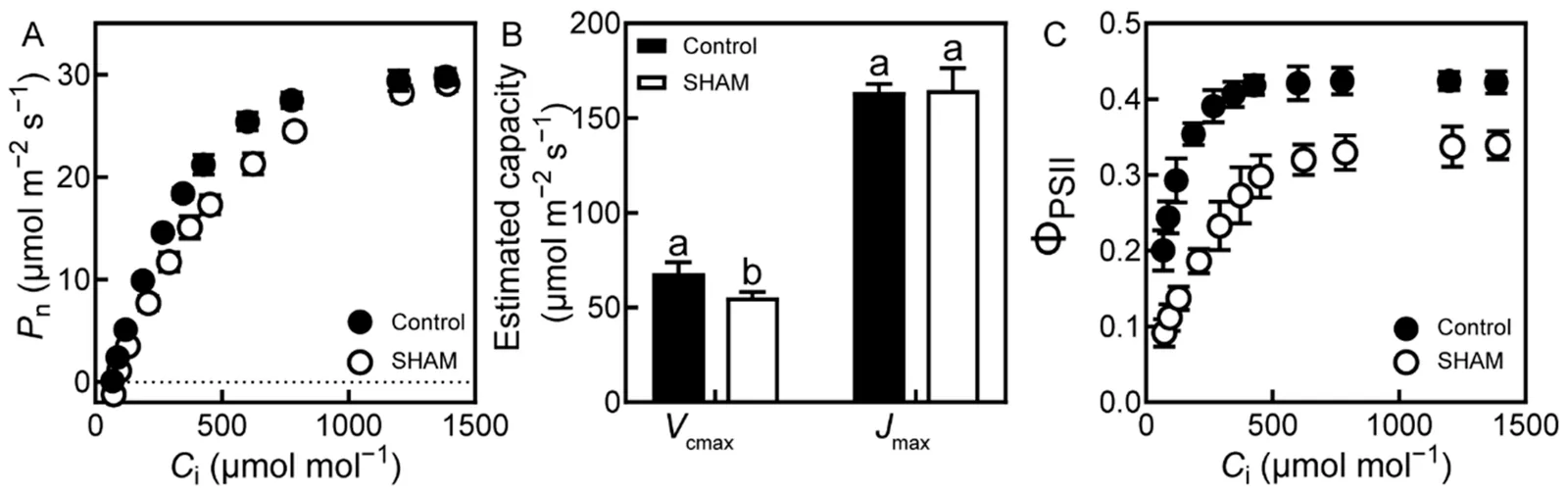
Effects of AOX pathway inhibition on CO_2_-response characteristics and estimated photosynthetic capacities in *Rumex* K-1 leaves. (**A**) Response of net photosynthetic rate (*P*_n_) to intercellular CO_2_ concentration (*C*_i_). (**B**) Maximum Rubisco carboxylation capacity (*V*_cmax_) and maximum electron-transport rate supporting RuBP regeneration (*J*_max_). (**C**) Response of the effective quantum yield of photosystem II (Φ_PSII_) to *C*_i_. Filled and open circles in panels (**A**) and (**C**) represent the control and SHAM treatments, respectively; black and white bars in panel (**B**) represent the corresponding treatments. Data are presented as means ± SE (*n* = 5). Different lowercase letters indicate significant differences between control and SHAM-treated leaves for the same parameter at *p* < 0.05.

In both treatments, *P*_n_ increased with increasing *C*_i_ and approached saturation at high *C*_i_ (Figure 5A). The difference in *P*_n_ between control and SHAM-treated leaves was greatest at low to intermediate *C*_i_ and progressively narrowed as *C*_i_ increased, with similar values observed at the two highest *C*_i_ levels (Figure 5A). SHAM treatment significantly reduced apparent maximum Rubisco carboxylation capacity (*V*_cmax_) by 18.8%, from 68.23 ± 2.54 to 55.43 ± 1.29 μmol m^−2^ s^−1^ (*p* = 0.002; Figure 5B), whereas maximum electron-transport rate supporting RuBP regeneration (*J*_max_) did not differ significantly between control and SHAM-treated leaves (163.84 ± 1.87 vs. 164.81 ± 5.13 μmol m^−2^ s^−1^, respectively; *p* = 0.863; Figure 5B). Φ_PSII_ also increased with increasing *C*_i_ before approaching a plateau, but remained lower in SHAM-treated leaves than in control leaves throughout the measured *C*_i_ range (Figure 5C).

**Figure 6:**
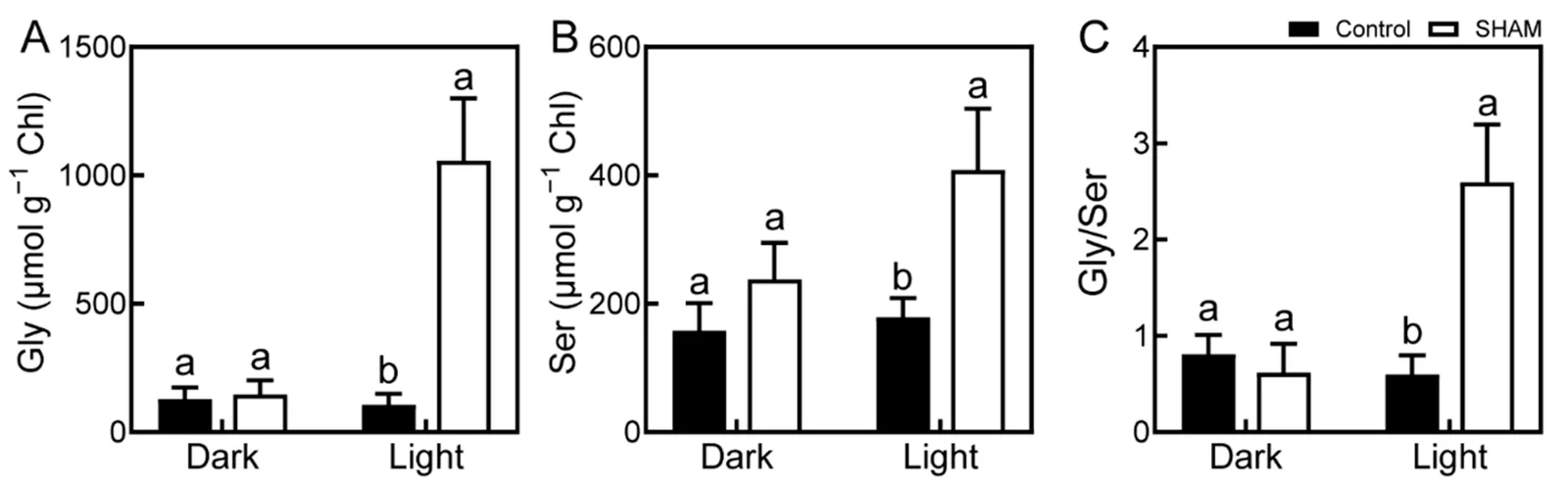
Effects of AOX pathway inhibition on glycine and serine accumulation in *Rumex* K-1 leaves under dark and light conditions. Glycine content [Gly; (**A**)], serine content [Ser; (**B**)], and the glycine-to-serine ratio [Gly/Ser; (**C**)] were determined in control and SHAM-treated leaves under dark and light conditions. Black and white bars represent the control and SHAM treatments, respectively. Data are presented as means ± SE (*n* = 5). Different lowercase letters indicate significant differences between treatments within each illumination condition at *p* < 0.05. Chl, chlorophyll.

In darkness, glycine and serine contents, as well as the Gly/Ser ratio, did not differ significantly between control and SHAM-treated leaves (Figure 6A–C). Under illumination, however, SHAM-treated leaves exhibited significantly higher glycine and serine contents and a higher Gly/Ser ratio than control leaves (Figure 6A–C; *p* < 0.05).

## 3. Discussion

Previous studies have established AOX as an important regulator coordinating mitochondrial redox metabolism with photosynthetic performance in illuminated leaves. Notably, the contribution of AOX to photosynthetic performance and photoprotection is particularly pronounced in C_3_ leaves under photorespiratory conditions and is diminished when photorespiration is suppressed [29,30]. These observations suggest that the photosynthetic function of AOX extends beyond maintaining redox homeostasis and is closely associated with mitochondrial processes supporting photorespiratory metabolism [20,29,30]. AOX inhibition significantly reduced CO_2_ assimilation in *Rumex* K-1 leaves. In SHAM-treated leaves, *P*_n_ declined significantly without a corresponding decrease in *g*_s_, whereas *C*_i_ increased, and *g*_m_ showed no detectable treatment-related decrease (Figure 1). Together, these responses argue against enhanced stomatal or mesophyll CO_2_ diffusion limitation as the primary cause of photosynthetic inhibition and instead support a predominantly non-stomatal limitation [31]. Although SHAM has also been reported to inhibit CO_2_ uptake and intracellular inorganic carbon accumulation in several green algae [32], raising the possibility that SHAM-sensitive carbon-acquisition processes may contribute to some photosynthetic responses, whether a comparable effect occurs in terrestrial C_3_ leaves such as *Rumex* K-1 remains unclear, given the distinct carbon-acquisition mechanisms of unicellular green algae and terrestrial C_3_ plants. The decline in *P*_n_ was accompanied by significant decreases in Φ_PSII_ and *J*_e_(PSII) (Figure 2A and Figure 3A), reflecting reductions in the effective quantum yield of PSII photochemistry and the apparent electron transport rate through PSII, respectively [33]. However, the increase in the Φ_PSII_/Φ_CO2_ ratio indicates that CO_2_ assimilation was inhibited to a greater extent than PSII electron transport, consistent with weakened coupling between photosynthetic electron transport and carbon fixation [33,34]. Electron-flux analysis further revealed decreases in both *J*_e_(PCR) and *J*_e_(PCO), the electron fluxes supporting the photosynthetic carbon reduction and photorespiratory carbon oxidation cycles, respectively [34,35]. Thus, AOX inhibition reduced electron use for both CO_2_ assimilation and photorespiratory carbon recycling. Overall, these photochemical responses are more consistent with feedback downregulation of photosynthetic electron transport in response to reduced downstream carbon-metabolic capacity than with a primary limitation originating from PSII photochemistry [36,37].

To further dissect the relationship between the photochemical changes induced by AOX inhibition and the limitation of CO_2_ assimilation, we used low-O_2_ and high-CO_2_ conditions to favour Rubisco carboxylation over oxygenation and thereby assess the contribution of photorespiration to the observed photosynthetic limitation [11,29]. Both treatments alleviated the SHAM-induced decline in CO_2_ assimilation, implicating photorespiratory metabolism in the photosynthetic limitation imposed by AOX inhibition. Under 2% O_2_, SHAM did not reduce apparent carboxylation efficiency (Figure 4). Likewise, *P*_n_ in SHAM-treated leaves progressively approached control values as *C*_i_ increased (Figure 5). The recovery of *P*_n_ despite incomplete restoration of Φ_PSII_ argues against impaired PSII photochemistry as the primary constraint on CO_2_ assimilation and instead points to a downstream limitation associated with photorespiratory carbon metabolism. A similar response was reported in the mitochondrial complex I-deficient CMSII mutant of *Nicotiana sylvestris*, in which reduced photosynthesis under ambient CO_2_ was substantially alleviated by high CO_2_ or low O_2_ [11]. In addition, *V*_cmax_ decreased under SHAM treatment, whereas *J*_max_ remained unchanged (Figure 5B), suggesting a greater effect on apparent Rubisco carboxylation capacity than on the maximum electron-transport capacity supporting RuBP regeneration. Together, these findings suggest that the major constraint on CO_2_ assimilation following AOX inhibition lies downstream of PSII and is associated with photorespiratory carbon metabolism, whereas the decline in photochemical efficiency likely reflects feedback downregulation in response to restricted carbon utilization [36,37].

The metabolite data provide a more specific biochemical link to this limitation. Upon illumination, SHAM increased both Gly and Ser, with the greater accumulation of Gly resulting in a significant increase in the Gly/Ser ratio (Figure 6), consistent with restricted rather than completely inhibited Gly-to-Ser conversion [30,38]. In mitochondria, the glycine decarboxylase complex (GDC) and serine hydroxymethyltransferase (SHMT) act in concert to convert two molecules of Gly into one molecule of Ser; during the GDC reaction, NAD^+^ serves as the electron acceptor and is reduced to NADH [38]. Sustained flux through the GDC–SHMT system therefore depends not only on the activities of GDC and SHMT but also on continuous reoxidation of GDC-derived NADH to regenerate NAD^+^ [39]. Although AOX does not directly oxidize NADH, AOX-mediated oxidation of ubiquinol helps maintain the ubiquinone pool in a sufficiently oxidized state to accept electrons from upstream NADH dehydrogenases, thereby supporting mitochondrial NADH oxidation and redox balance [40]. Under illumination, mitochondrial Gly oxidation and chloroplast photosynthesis both generate reducing equivalents that must be balanced across cellular compartments, with metabolite shuttles such as the Mal/OAA shuttle contributing to their redistribution [41,42]. AOX inhibition may therefore constrain mitochondrial reductant oxidation when shuttle-mediated redistribution and the remaining respiratory pathways are insufficient to compensate, potentially shifting the mitochondrial NAD(H) pool toward a more reduced state, limiting NAD^+^ availability, and restricting sustained GDC–SHMT turnover [43].

The Gly/Ser response to AOX restriction, however, varies among experimental systems. Under high light, SHAM increased the Gly/Ser ratio in C_3_ leaves but had little effect in C_4_ leaves, where photorespiration is strongly suppressed [30], consistent with a close association between this response and photorespiratory activity. The magnitude of the response is also likely to depend on the capacity of other redox-processing routes to compensate. Alternative NAD(P)H dehydrogenases may broaden the routes available for NAD(P)H oxidation; however, because they feed electrons into the ubiquinone pool upstream of AOX, they cannot directly substitute for AOX-mediated ubiquinol oxidation, and their ability to support NAD(P)H oxidation during AOX restriction therefore depends on sufficient downstream oxidation of ubiquinol through the remaining respiratory pathways [44,45,46]. Previous measurements in the same *Rumex* K-1 system showed measurable AOX pathway capacity in darkness and marked increases in both AOX pathway capacity and NADP-MDH initial activity following intense-light exposure [12]. Together with the absence of a SHAM-induced Gly/Ser change in darkness and its pronounced increase under illumination in the present study, these findings are consistent with a greater requirement for mitochondrial and interorganellar reductant processing during active photorespiration. Genetic studies further support this interpretation: enhanced GDC capacity, achieved by increasing H-protein abundance or L-protein activity, reduces Gly accumulation and enhances photosynthetic performance, whereas reduced GDC capacity impairs glycine oxidation, causes marked Gly accumulation during illumination, and decreases photosynthesis [47,48,49,50].

Restricted Gly-to-Ser conversion provides an additional metabolic explanation for the decline in CO_2_ assimilation following AOX inhibition. The photorespiratory cycle recovers carbon from 2-phosphoglycolate (2-PG) generated by Rubisco oxygenation and returns it to the Calvin–Benson cycle as 3-phosphoglycerate (3-PGA). A reduction in photorespiratory turnover could therefore decrease the recovery of 2-PG-derived carbon as 3-PGA and thereby constrain the replenishment of Calvin–Benson-cycle intermediates [51]. Findings in rice, where mesophyll-targeted reduction in GDC H-protein abundance led to Gly accumulation and reduced CO_2_ assimilation under photorespiratory conditions, further support the importance of sustained mitochondrial Gly metabolism for photosynthetic carbon assimilation [52]. Accumulation of photorespiratory intermediates may impose an additional constraint on carbon fixation by altering Rubisco activation. Glyoxylate, for example, has been reported to affect the Rubisco activation state [53,54], and suppression of glycolate oxidase in rice similarly reduced Rubisco activase expression and the activation state of Rubisco [55]. These observations provide a plausible parallel mechanism by which altered photorespiratory metabolism could contribute to the apparent Rubisco-side limitation identified in the CO_2_-response analysis. SHAM-sensitive glycolate-oxidation systems have also been reported in algae and isolated chloroplast preparations [56,57]; although these differ from the classical peroxisomal glycolate oxidase system of higher C_3_ plants, a contribution of altered glycolate/glyoxylate metabolism to the SHAM response cannot be excluded.

**Figure 7:**
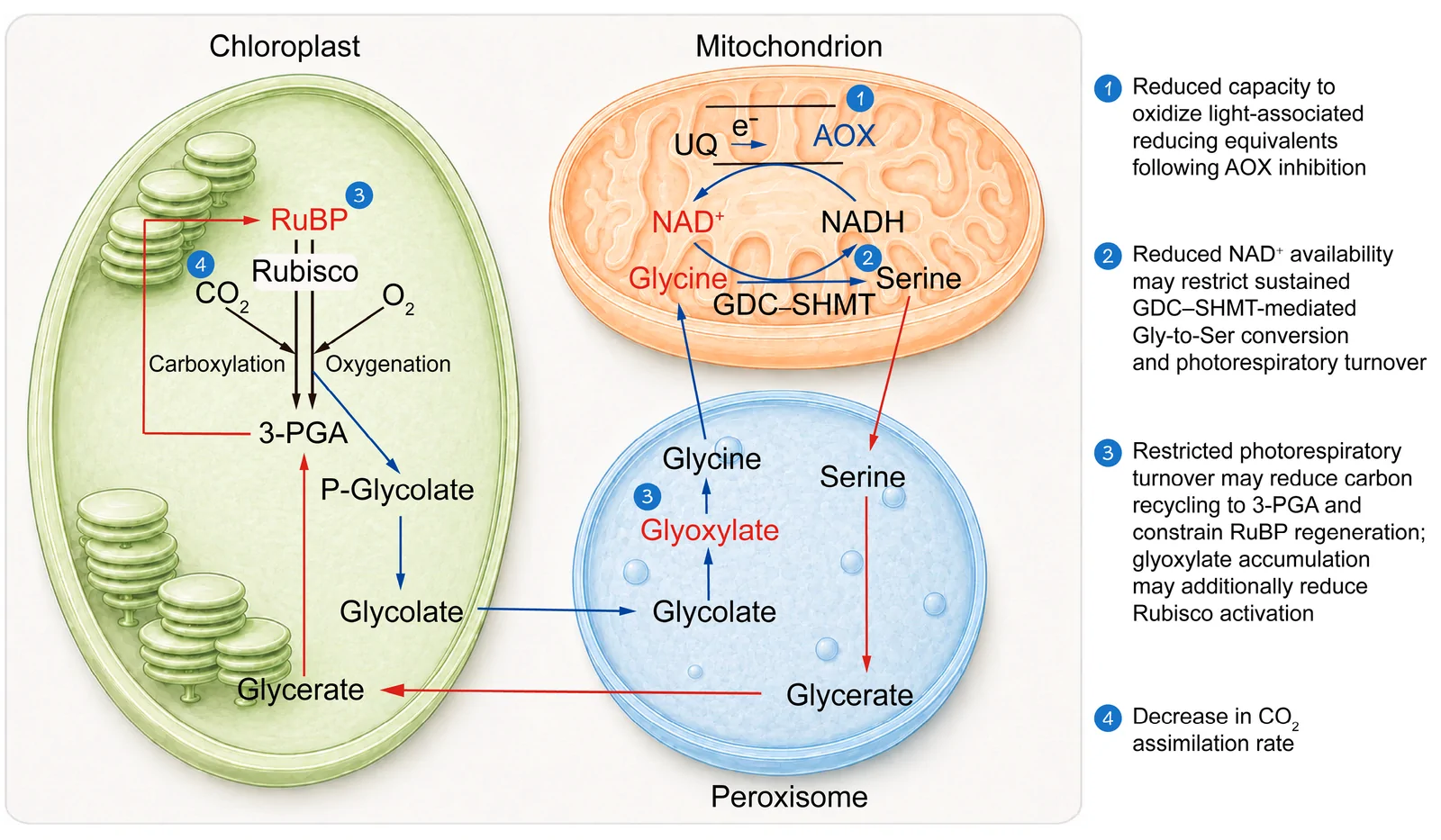
Proposed mechanism by which inhibition of the mitochondrial alternative oxidase pathway suppresses photosynthesis in *Rumex* K-1 leaves. Inhibition of AOX may reduce the mitochondrial capacity to dissipate excess reducing equivalents, thereby restricting NAD^+^ regeneration (1). Reduced NAD^+^ availability may restrict sustained glycine decarboxylase (GDC)–serine hydroxymethyltransferase (SHMT)-dependent Gly-to-Ser conversion, thereby limiting photorespiratory turnover (2). Consequently, restricted photorespiratory turnover may reduce the recycling of carbon derived from 2-phosphoglycolate to glycerate and 3-phosphoglycerate (3-PGA), thereby constraining ribulose-1,5-bisphosphate (RuBP) regeneration in the Calvin–Benson cycle. In parallel, glyoxylate accumulation may reduce the activation state of Rubisco (3). Together, these effects contribute to the decrease in CO_2_ assimilation (4). Blue arrows indicate the forward photorespiratory pathway and mitochondrial electron transfer, red arrows indicate the return of photorespiratory carbon to the chloroplast and RuBP regeneration, and black arrows indicate Rubisco-catalyzed carboxylation and oxygenation reactions. UQ, ubiquinone; AOX, alternative oxidase; GDC, glycine decarboxylase; SHMT, serine hydroxymethyltransferase; RuBP, ribulose-1,5-bisphosphate; 3-PGA, 3-phosphoglycerate.

Collectively, our results support a mechanistic model in which AOX inhibition may restrict mitochondrial reductant-oxidation capacity, constrain photorespiratory Gly-to-Ser turnover and carbon recycling, and ultimately reduce CO_2_ assimilation (Figure 7). These findings suggest that the photosynthetic limitation imposed by AOX inhibition arises primarily from disrupted coordination between mitochondrial redox homeostasis and photorespiratory carbon metabolism rather than from restricted photosynthetic electron transport alone.

## 4. Materials and Methods

### 4.1. Plant Material and Experimental Design

*Rumex* K-1 (*R. patientia* × *R. tianschanicus*) was grown in pots with drainage holes filled with vermiculite and supplied with Hoagland nutrient solution [58]. Plants were maintained in a greenhouse at 20–28 °C, where daytime PPFD was approximately 600 μmol photons m^−2^ s^−1^. Two plants were maintained per pot. Healthy, fully expanded functional leaves at a comparable developmental stage were selected from the outer, light-facing part of each plant, where they were fully exposed to incident greenhouse light and were not appreciably shaded by neighbouring leaves.

A 100 mM salicylhydroxamic acid (SHAM; Sigma, St. Louis, MO, USA) stock solution was prepared by dissolving 0.766 g SHAM in 20 mL absolute ethanol (Sigma, St. Louis, MO, USA), followed by the addition of 30 mL distilled water. The stock solution was diluted 100-fold with distilled water to obtain a final SHAM concentration of 1 mM. The corresponding control solution contained the same final concentration of ethanol as the SHAM working solution. The 1 mM SHAM concentration was selected on the basis of previous inhibitor-response and isolated-chloroplast control experiments conducted in our laboratory, which showed substantial inhibition of alternative oxidase (AOX) pathway capacity without a detectable direct effect on the chloroplast photochemical parameters examined [12].

Leaves were excised at the petiole, and the petiole ends were immediately re-cut under either the control solution or 1 mM SHAM to minimize air entry into the xylem and maintain hydraulic continuity. Thereafter, only the cut petiole ends remained in the respective solutions, whereas the leaf blades were exposed to air throughout the pretreatment. Leaves were allowed to take up the solutions for 8 h under low light at approximately 20 μmol photons m^−2^ s^−1^ before subsequent treatments and measurements. For each treatment, five leaves, each collected from a different plant in a different pot, were used as independent biological replicates.

### 4.2. Gas-Exchange Measurements

Net CO_2_ assimilation rate (*P*_n_), stomatal conductance (*g*_s_), and intercellular CO_2_ concentration (*C*_i_) were measured using a CIRAS-2 portable photosynthesis system (PP Systems, Amesbury, MA, USA). All gas-exchange measurements were conducted under controlled cuvette conditions with leaf temperature maintained at 25 °C by the instrument temperature-control system; actinic irradiance was supplied by the built-in light source, reference CO_2_ was regulated by the instrument, and measurements were made under ambient O_2_ (21%, *v*/*v*) unless otherwise specified. For light-response measurements, reference CO_2_ was maintained at 400 μmol mol^−1^ and PPFD was decreased stepwise in the following sequence: 1600, 1200, 800, 600, 400, 300, 200, 150, 100, and 50 μmol photons m^−2^ s^−1^. At each irradiance, gas-exchange variables were allowed to stabilize before values were recorded and the next PPFD step was applied.

For the *P*_n_–*C*_i_ response curves, leaf temperature was maintained at 25 °C, PPFD was fixed at the light-saturating level of 1000 μmol photons m^−2^ s^−1^, and reference CO_2_ was adjusted sequentially to 1600, 1400, 1000, 800, 600, 400, 300, 200, 100, and 50 μmol mol^−1^ under 21% O_2_. The corresponding *P*_n_ and *C*_i_ values were used to construct the *P*_n_–*C*_i_ response curves. Apparent carboxylation efficiency (CE) was calculated as the slope of the initial, approximately linear portion of each *P*_n_–*C*_i_ response curve [59]. For each biological replicate, the maximum Rubisco carboxylation capacity (*V*_cmax_) and maximum electron-transport rate supporting RuBP regeneration (*J*_max_) were estimated using the C_3_ biochemical model of Farquhar et al. [60] and the fitting procedure of Sharkey et al. [61].

To assess the effect of O_2_ availability on CE, *P*_n_–*C*_i_ response curves were additionally measured under atmospheres containing either 21% or 2% (*v*/*v*) O_2_. The respective O_2_ atmospheres were established by supplying premixed standard gases containing 21% or 2% O_2_ through the air inlet of the CIRAS-2 system. During these measurements, the reference CO_2_ concentration was regulated by the CIRAS-2 system. The use of 2% O_2_ as the low-O_2_ treatment followed the experimental design described by Zhang et al. (2017) [30].

### 4.3. Measurement of Chlorophyll Fluorescence

Chlorophyll fluorescence was measured using an FMS-2 pulse-modulated chlorophyll fluorometer (Hansatech Instruments, King’s Lynn, UK). Under each measurement condition, steady-state fluorescence (*F*_s_) was recorded following 30 s of actinic illumination. The maximum fluorescence in the light-adapted state (*F*_m_*′*) was then determined by applying a saturating pulse of 8000 μmol photons m^−2^ s^−1^ for 0.7 s. The effective quantum yield of photosystem II (Φ_PSII_) was calculated as (*F*_m_′ − *F*_s_)/*F*_m_′ according to Genty et al. (1989) [33].

The apparent quantum yield of net CO_2_ assimilation (Φ_CO2_) and the Φ_PSII_/Φ_CO2_ ratio were calculated from the corresponding gas-exchange and chlorophyll-fluorescence measurements obtained under the same measurement conditions, following Oberhuber et al. (1993) [62]. Mesophyll conductance (*g*_m_) was estimated from paired *P*_n_–*C*_i_ and Φ_PSII_–*C*_i_ measurements obtained from the same leaves using the variable-*J* method [63,64]. For each biological replicate, *P*_n_ and Φ_PSII_ were linearly interpolated to a common *C*_i_ of 550 μmol mol^−1^, at which all leaves satisfied the reliability criterion of the variable-*J* approach. Chloroplastic CO_2_ concentration (*C*_c_) was estimated using a CO_2_ compensation point in the absence of mitochondrial respiration (Γ*) of 42.75 μmol mol^−1^ at 25 °C [65], with day respiration estimated from dark respiration according to a previous study [66], and *g*_m_ was calculated as *P*_n_/(*C*_i_ − *C*_c_). The electron flux through PSII [*J*_e_(PSII)] was estimated from Φ_PSII_ and incident PPFD, assuming a leaf absorptance of 0.84 and equal partitioning of absorbed excitation energy between PSI and PSII. Electron fluxes associated with the photosynthetic carbon reduction cycle [*J*_e_(PCR)] and the photorespiratory carbon oxidation cycle [*J*_e_(PCO)] were estimated from the combined gas-exchange and chlorophyll-fluorescence data according to Valentini et al. (1995) [34].

### 4.4. Glycine and Serine Quantification

Following the 8-h uptake pretreatment, control and SHAM-treated detached leaves were subjected to either complete darkness or light at a photosynthetic photon flux density of 800 μmol photons m^−2^ s^−1^ for an additional 2 h. At the end of the treatment, leaf tissue was immediately sampled, weighed, frozen in liquid nitrogen, and used for free amino acid analysis.

Free amino acids were extracted according to Igarashi et al. (2006) [67]. Briefly, leaf tissue was extracted with 80% (*v*/*v*) ethanol at 80 °C. The extracts were evaporated to dryness, and the residues were reconstituted in 0.02 mol L^−1^ HCl (Sigma, St. Louis, MO, USA). Glycine (Gly) and serine (Ser) were quantified using a Hitachi 835-50 automated amino acid analyzer (Hitachi, Tokyo, Japan), which performed amino acid separation by cation-exchange chromatography, post-column derivatization with ninhydrin (Sigma, St. Louis, MO, USA), and spectrophotometric detection.

At the time of amino acid sampling, a parallel subsample was collected from the same leaf for chlorophyll determination. Chlorophyll was extracted with 80% (*v*/*v*) acetone (Sigma, St. Louis, MO, USA), and absorbance was measured using a UV-2600i ultraviolet–visible (UV–Vis) spectrophotometer (Shimadzu Corporation, Kyoto, Japan) at 645, 663, and 750 nm. The absorbance values at 645 and 663 nm were corrected using the absorbance at 750 nm, and total chlorophyll content was calculated according to Porra et al. (1989) [68]. Gly and Ser contents were normalized to the total chlorophyll content of the corresponding leaf sample and expressed as μmol g^−1^ chlorophyll. The Gly/Ser ratio was subsequently calculated.

### 4.5. Statistical Analysis

Data are presented as means ± standard errors (SE) of five independent biological replicates per treatment. Differences between control and SHAM-treated leaves were evaluated using two-sided unpaired Student’s *t*-tests. For experiments conducted under different O_2_ concentrations or illumination conditions, comparisons were performed between control and SHAM-treated leaves separately within each condition; no direct statistical comparisons were made between O_2_ concentrations or between dark and light treatments. Differences were considered statistically significant at *p* < 0.05. Graphs were generated and statistical analyses were performed using GraphPad Prism version 11 (GraphPad Software, Boston, MA, USA).

## Data Availability

The data presented in this study are available in the article. Further inquiries can be directed to the corresponding author.
